# Changes in the Interstitial Cells of Cajal and Immunity in Chronic Psychological Stress Rats and Therapeutic Effects of Acupuncture at the Zusanli Point (ST36)

**DOI:** 10.1155/2016/1935372

**Published:** 2016-08-03

**Authors:** Mucang Liu, Shenglin Zhang, Yue Gai, Mingzheng Xie, Qinghui Qi

**Affiliations:** Department of General Surgery, The First Affiliated Hospital of Dalian Medical University, Liaoning 116011, China

## Abstract

Now, chronic psychological stress (CPS) related diseases are increasing. Many CPS patients have gastrointestinal complaints, immune suppression, and immune imbalance. Increasing evidence is indicating that acupuncture (AP) at the Zusanli point (ST36) can alleviate functional gastrointestinal disorders (FGID), immune suppression, and immune imbalance. However, few studies have investigated the potential mechanisms. In this study, CPS rat models were established, and electroacupuncture (EA) at ST36 was done for CPS rats. Daily food intake, weight, intestinal sensitivity, the morphology of interstitial cell of Cajal (ICC) in the small intestine, and serum indexes were measured. The study found that, in CPS rats, EA at ST36 could improve food intake, weight, visceral hypersensitivity, and immunity; in CPS rats, in small intestine, the morphology of ICCs was abnormal and the number was decreased, which may be part causes of gastrointestinal motility dysfunction. EA at ST36 showed useful therapeutic effects. The mechanisms may be partially related to its repairing effects on ICCs damages; in CPS rats, there were immune suppression and immune imbalance, which may be part causes of visceral hypersensitivity. EA at ST36 showed useful therapeutic effects. The mechanisms may be partially related to its regulation on immunity.

## 1. Introduction

With the changes of the social environment and ways of life, job stress is increasing and the pace of life is accelerating. Meanwhile, chronic psychological stress (CPS) related diseases are growing rapidly.

The digestive system is very vulnerable to the influence of CPS. Functional gastrointestinal disorders (FGID) are very common in daily clinical practice, and they are characterized by disturbances in the motility patterns and/or visceral hypersensitivity [1, 2]. CPS plays an important role in the development and exacerbation of symptoms in FGID [3]. At present, its pathogenesis of stress-related FGID has not been studied very clearly. Generally, it is common to use drugs for FGID including antacids, prokinetic agents, antianxiety or antidepression drugs, digestants, and anti-HP treatment. But there is no specific remedy [4]. Also, studies have shown that CPS is associated with immune suppression and immune imbalance [5, 6].

Acupuncture (AP), a traditional Chinese medicine, has garnered significant attention due to its role in regulation of gastrointestinal function. In the clinic, growing evidences have shown that it could alleviate FGID [7–9]. Also, studies have shown that AP could improve immunosuppression in patients [10, 11]. However, few studies have investigated the potential mechanisms underlying these effects.

The interstitial cell of Cajal (ICC) exhibits a highly branched morphology and forms unique network in the gastrointestinal tract (GI). ICCs serve as electrical pacemakers, active propagation pathways for slow waves, and mediators of enteric motor neurotransmission. They play an important role in generating and regulating gastrointestinal motility [12]. The enteric nervous system (ENS), ICCs, and smooth muscle cells (SMCs) connect to form a network structure, which is the basic functional unit of gastrointestinal motility. Studies have shown that the amount of ICCs is abnormal or decreased in gastrointestinal motility dysfunction diseases, or the neurotransmission of the ENS-ICCs-SMC network may be decreased [13–15]. This study is seeking to identify changes in ICCs in CPS rats and the therapeutic effects of electroacupuncture (EA) at ST36.

Visceral hypersensitivity and gastrointestinal inflammation are important pathophysiological factors for gastrointestinal motility dysfunction diseases, and they closely correlate with immunity [16–19]. In this study, we were aiming to characterize the immune response in CPS rats and the therapeutic effects of EA at ST36.

Repeated exposure to water avoidance stress has been shown to successfully establish a CPS rat model for sustained visceral hyperalgesia [20, 21]. In this study, CPS rat models were established. Food intake, weight, intestinal sensitivity, ICC of small intestine, and serum immune indexes were measured, and therapeutic effects of EA at ST36 were investigated.

## 2. Materials and Methods

### 2.1. Materials

Thirty seven-week-old male Wistar rats were provided by the experimental animal center of Dalian Medical University. The rats were maintained in a normal light-dark cycle, housed 3-4 rats per cage, and provided with free access to food and water. All protocols were approved by the Ethics Committee of The First Affiliated Hospital of Dalian Medical University.

### 2.2. Animal Grouping, Model Preparation, AP, and Specimen Collection (Figure 1)

The CPS rat models were established by the method of Bradesi et al. [20]. According to a random number table method, the rats were grouped as follows: the control group: 6 rats; the model group: 12 rats; the AP group: 6 rats; the sham-AP group: 6 rats. All rats except those in the control group were exposed to 10 days of water avoidance stress. The test apparatus consisted of Plexiglas tanks (50 cm length × 25 cm width × 25 cm height) with a block (10 × 8 × 8 cm) affixed to the center of the floor. The tank was filled with fresh room temperature water (25°C) to within 1 cm of the top of the block. The rats were placed on the block for 1 h daily for 10 consecutive days. In the control group, rats were similarly placed on the same platform in a waterless container for 1 h daily for 10 days. This well-characterized test represents a potent psychological stressor associated with large increases adrenocorticotropic hormone and corticosterone within 30 min [21].

At day 11, rats in the control group were housed separately for one day, and daily food intake (24 h) was measured. At day 12, the rats were fasted starting at 3 p.m. but provided free access to water. At day 13, the rats were weighed and intestinal sensitivity was evaluated. Then, the rats were sacrificed under anesthesia. Blood was collected from the inferior vena cava, and the proximal jejunum was cut and collected. The blood was centrifuged at 3000 r/min for 15 min, and the serum was segregated for subsequent analyses. The proximal jejunum was washed quickly with normal saline and then dissected into 1-2 cm fragments, fixed in 4% paraformaldehyde, and placed in the refrigerator. At day 11, 6 rats in the model group were selected according to the random number table method. The rats were processed the same way as the control group.

At day 11, rats in the model group (the remaining 6 rats), AP group, and sham-AP group were fed for another 15 days. In the AP group, every day, rats underwent binding and EA at ST36 for 20 minutes between 7 a.m. and 9 a.m. The acupoint was located at the posterior and lateral side of the knee joint, 5 mm below capitulum fibulae [22]. The AP point was punctured to a depth of 5 mm, and the needle was then connected to an EA apparatus to apply a 0.1–04 mA (based on the tolerance of rat) and 2/15 Hz electric current. In the sham-AP group, sham-AP was executed by the same method, but the needles were inserted at a nonchannel acupoint located 10 mm lateral inferior to ST36. In the model group, rats did not undergo EA but underwent binding every day.

At day 24, in each group, rats were housed separately for one day, and daily food intake (24 h) was measured. At day 25, the rats were fasted starting at 3 p.m. but provided free access to water. At day 26, each rat was weighed and intestinal sensitivity was evaluated. Then, the blood and jejunum were processed as described above.

### 2.3. Intestinal Sensitivity

Abdominal withdrawal reflex (AWR) in response to acute visceral pain was used to assess the intestinal sensitivity following the method of Al-Chaer et al. [23]. Acute visceral pain was evoked by colorectal distention (CRD).

*CRD*. The distention was applied using angioplasty balloons (length, 25.0 mm; diameter, 3.0 mm) inserted rectally into the descending colon of mildly sedated rats (Brevital, 5 mL, 1% intraperitoneally) and secured by taping the attached tubing to the rat's tail. The rats were then housed in small Lucite cubicles (20 × 8 × 8 cm) on an elevated Plexiglas and allowed to wake up and adapt (1 hour). Measurement of the AWR consisted of visual observation of the animal response to graded CRD (20, 40, 60, and 80 mmHg) by blinded observers and assignment of an AWR score. The rats were given CRD for 20 seconds every 4 minutes. To achieve an accurate measure, the distention was repeated 5 times for each intensity. The data for each animal was averaged for analysis. The results obtained were compared across groups.

*The Score of AWR*. 0 indicates no behavioral response to CRD; 1, brief head movement followed by immobility; 2, contraction of abdominal muscles; 3, lifting of abdomen; 4, body arching and lifting of pelvic structures. Behavioral measurements were reproduced by 2 different blinded observers. CRD was applied in increments of 10 mmHg starting at 10 mmHg.

### 2.4. Deep Muscular Plexus of ICCs in Intestinal Tissue Using Confocal Laser Scanning Microscopy

A part of proximal jejunum was cut into 1-2 cm fragments, fixed in 4% paraformaldehyde, and placed in the refrigerator at 4°C overnight. The tissues were dissected with an anatomic microscope to strip the mucosa and submucosa as one layer and retain the whole intestinal muscularis.

Next, (1) the specimens were incubated in 0.5% Triton-X in 0.05 mol/L Tris-HCl buffer (pH 7.6) solution at 37°C for 4 h; (2) after rinsing twice in PBS, specimens were incubated for 1 h at room temperature in 1% BSA to prevent nonspecific binding; (3) after rinsing twice in PBS, specimens were incubated for 48 h at 4°C in rabbit anti-rat c-kit polyclonal antibody (c-19, Santa Cruz Biotech, USA); (4) specimens were rinsed twice and incubated 2 h at 4°C in the dark in Cy3-mouse anti-rabbit IgG antibody (Santa Cruz Biotech, USA); (5) specimens were embedded in fluorescence mounting medium and examined under a confocal laser scanning microscope [24].

In step (3), the control group was incubated in only antibody diluent instead of antibody.

Specimens were observed using a TCS-SP2 laser scanning confocal microscope (Leica, Germany) with immersion objectives (×40, numerical aperture 1.3). Specimens were excited using a krypton/argon laser with excitation and barrier filters set for fluorophores according to its excitation-emission spectra (Cy3 = 550 nm). The emitted light was measured by a photomultiplier tube and converted into a digital pixelated image by an analog-to-digital converter. The detection pinhole was set for use with different objectives accordingly. C-kit positive fluorescence was red. Six specimens were observed in each group, and three fields of vision were observed randomly in each specimen. Three-dimensional images were constructed and integrated optical density (IOD) was performed by the Leica confocal software.

### 2.5. The Serum IgG, IgM, IL-2, and IL-6 Levels

The serum immunoglobulin G (IgG) and immunoglobulin M (IgM) levels were measured using commercially available sandwich ELISA kits (Xiang Sheng Biotechnology, Shanghai, China) according to the manufacturer's instructions. Absorbance was read at 492 nm. The serum interleukin-2 (IL-2) and interleukin-6 (IL-6) levels were measured using commercially available sandwich ELISA kits (Boster Biotechnology, Wuhan, China) in accordance with the manufacturer's instructions. Absorbance was read at 450 nm.

The measurements of weight and food intake, the assessment of intestinal sensitivity, and the measurement of ICCs and serum immune indexes were performed by blinded researchers.

## 3. Statistical Analysis

The food intake, weight, scores of AWR, IOD of ICCs, and immune indexes data were analyzed by a commercial software package (SPSS13.0). Data was expressed as the mean ± standard error. Differences between two groups were compared by the *t*-test. Differences between multigroup were compared by the one-way ANOVA; then differences between two groups were compared by S-N-K test, and *P* < 0.05 denoted the difference possessing statistical significance.

## 4. Results

### 4.1. The Comparison of the Control Group and Model Group (Day 11 and Day 13)

#### 4.1.1. Food Intake (Day 11) and Weight (Day 13)

**Figure 2:**
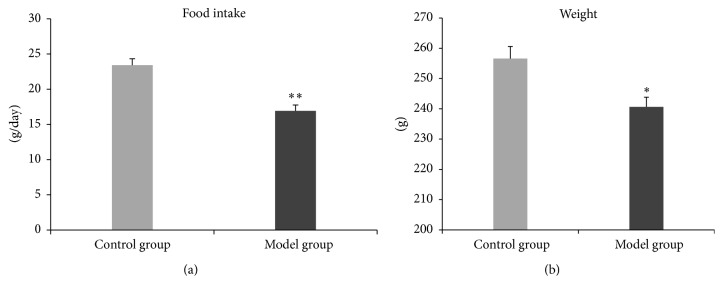
Food intake and weight. In the model group, daily food intake is significantly lower than that in the control group (*t* = −5.360, *P* < 0.01) (a); weight is significantly lower also (*t* = −3.144, *P* < 0.05) (b). ^*∗*^ *P* < 0.05; ^*∗∗*^ *P* < 0.01.

In the model group, food intake and weight were significantly lower than those in the control group (Figure 2).

#### 4.1.2. Scores of AWR (Day 13)

**Figure 3:**
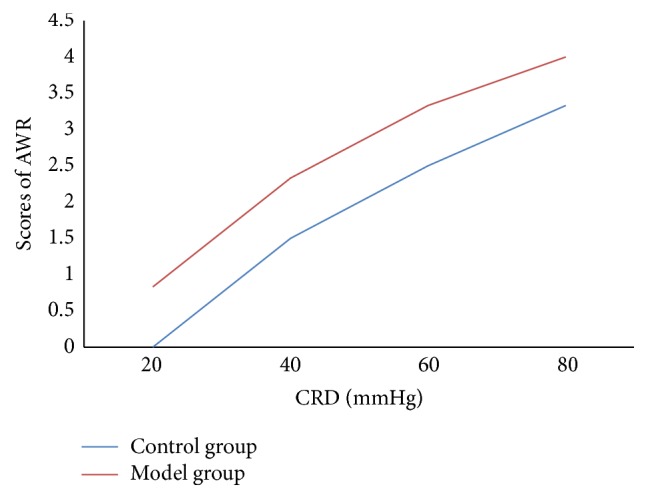
Scores of AWR. In the model group, when the pressure of CRD was 20, 40, 60, or 80 mmHg, score of AWR is significantly higher than that in the control group (*t* = 5.00, *P* < 0.01; *t* = 2.71, *P* < 0.05; *t* = 2.71, *P* < 0.05; *t* = 3.16, *P* < 0.05).

In the model group, when the pressure of CRD was 20, 40, 60, or 80 mmHg, score of AWR was significantly higher than that in the control group (Figure 3).

#### 4.1.3. Deep Muscular Plexus of ICCs in Intestinal Tissue Using Confocal Laser Scanning Microscopy (Day 13)

**Figure 4:**
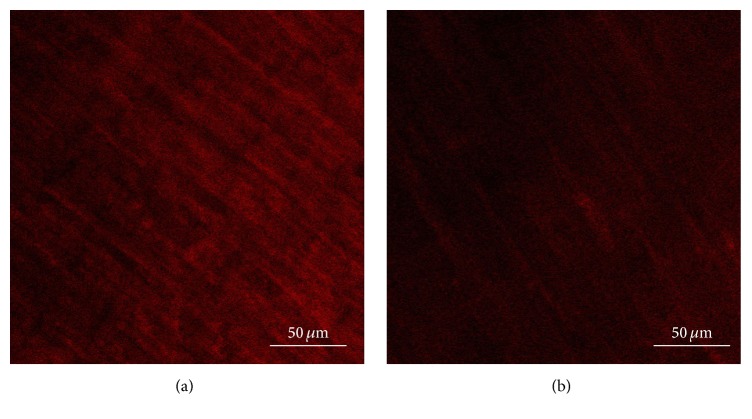
ICCs in intestinal tissue. The control group (a) shows that ICCs are spindle-shaped cells with 2-3 synapses; they are connected to each other by synapses and form network structure. The model group (b) shows that the number of ICCs and the synapses of ICCs are decreased, and the network integrity is damaged.

The control group (Figure 4(a)) shows that ICCs were spindle-shaped cells with 2-3 synapses; they were connected to each other by synapses and formed network structure.

**Figure 5:**
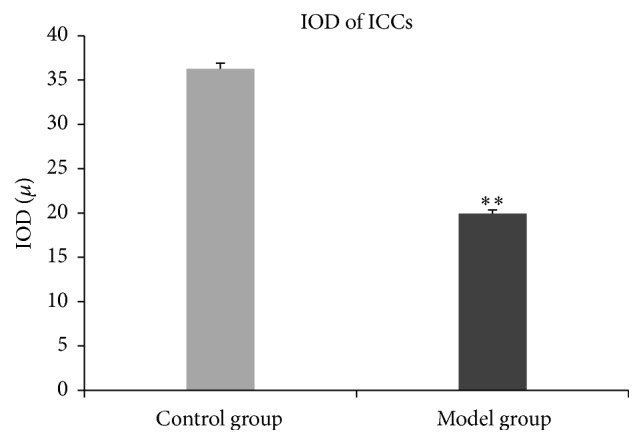
IOD of ICCs. In the model group, the IOD of ICCs is significantly lower than that in the control group (*t* = −21.45, *P* < 0.01). ^*∗∗*^ *P* < 0.01.

The model group (Figure 4(b)) shows that the number of ICCs and the synapses of ICCs were decreased, and the network integrity was damaged. The IOD of ICCs was significantly lower than that in the control group (Figure 5).

#### 4.1.4. Serum IgG, IgM, IL-2, and IL-6 Levels (Day 13)

**Figure 6:**
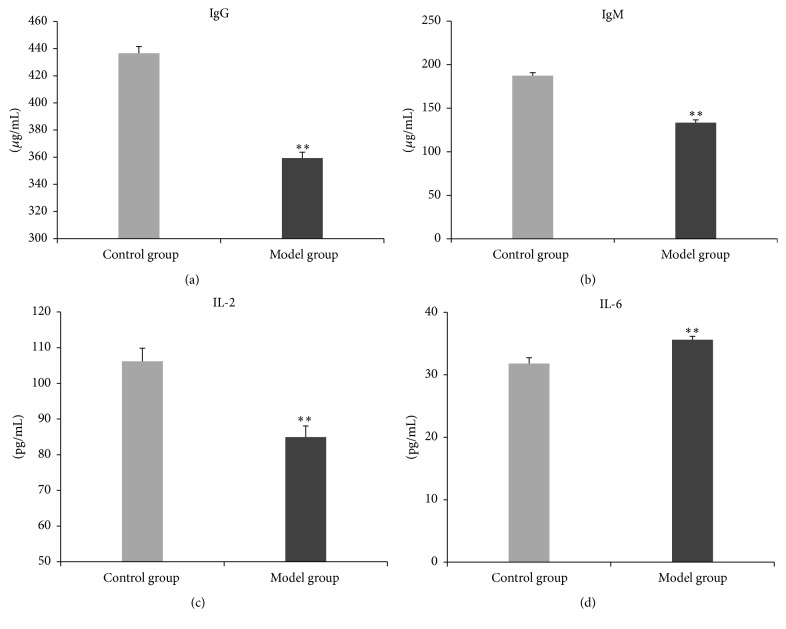
Serum IgG, IgM, IL-2, and IL-6 levels. In the model group, the serum IgG, IgM, and IL-2 levels are significantly lower than those in the control group ((a) *t* = −11.67, *P* < 0.01; (b) *t* = −11.26, *P* < 0.01; (c) *t* = −4.44, *P* < 0.01). The serum IL-6 level is significantly higher than that in the control group ((d) *t* = 5.23, *P* < 0.01). ^*∗∗*^ *P* < 0.01.

In the model group, the serum IgG, IgM, and IL-2 levels were significantly lower than those in the control group (Figures 6(a), 6(b), and 6(c)), and the serum IL-6 level was significantly higher than that in the control group (Figure 6(d)).

### 4.2. The Comparison of the Model, AP, and Sham-AP Groups (Day 24 and Day 26)

#### 4.2.1. Food Intake (Day 24) and Weight (Day 26)

**Figure 7:**
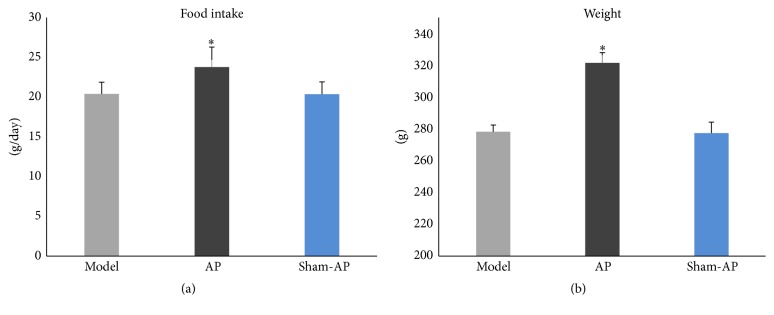
Food intake and weight. There is significant difference in daily food intake among the three groups (*F* = 6.33, *P* < 0.05) (a). In the AP group, food intake is significantly higher than that in the model group (*P* < 0.05), and there is no significant difference between the model group and the sham-AP group; there is significant difference in weight among the three groups (*F* = 108.12, *P* < 0.01) (b). In the AP group, weight is significantly higher than that in the model group (*P* < 0.05), and there is no significant difference between the model group and the sham-AP group. ^*∗*^ *P* < 0.05.

In the AP group, food intake (Figure 7(a)) and weight (Figure 7(b)) were significantly higher than those in the model group, and there was no significant difference between the model group and the sham-AP group.

#### 4.2.2. Scores of AWR (Day 26)

**Figure 8:**
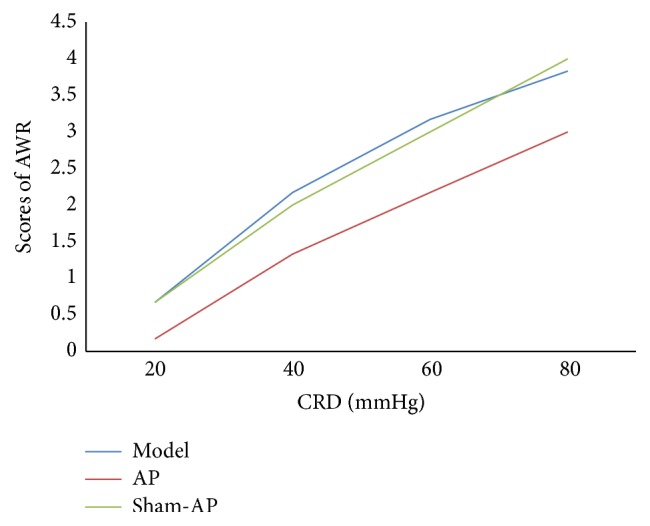
Scores of AWR. When pressure of CRD was 20 mmHg, there is no significant difference in scores of AWR among the three groups (*F* = 2.14, *P* > 0.05); when pressure of CRD was 40, 60, or 80 mmHg, there is significant difference in scores of AWR among the three groups (*F* = 8.08, *P* < 0.01; *F* = 15.50, *P* < 0.01; *F* = 31.00, *P* < 0.01). In the AP group, score of AWR is significantly lower than that in the model group (*P* < 0.05), and there is no significant difference between the model group and the sham-AP group.

When the pressure of CRD was 20 mmHg, scores of AWR were not significantly different among the model group, AP group, and sham-AP group; when the pressure of CRD was 40, 60, or 80 mmHg, in the AP group, score of AWR was significantly lower than that in the model group, and there was no significant difference between the model group and the sham-AP group (Figure 8).

#### 4.2.3. Deep Muscular Plexus of ICCs in Intestinal Tissue Using Confocal Laser Scanning Microscopy (Day 26)

**Figure 9:**
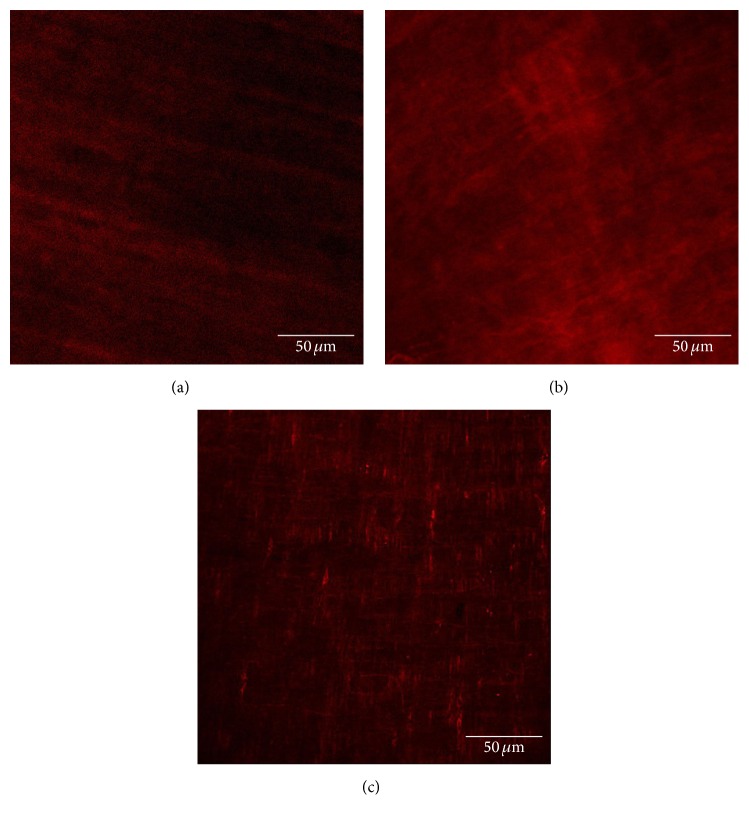
ICCs in intestinal tissue. The model group (a) shows that the number of ICCs and the synapses of ICCs are low, and the network integrity is damaged. The AP group (b) shows that the number of ICCs and the synapses of ICCs are significantly increased. ICCs are connected to each other by synapses and form network structure. The sham-AP group (c) shows that the number of ICCs and the synapses of ICCs are low, and the network integrity is damaged.

The model group (Figure 9(a)) shows that the number of ICCs and the synapses of ICCs were low, and the network integrity was damaged.

**Figure 10:**
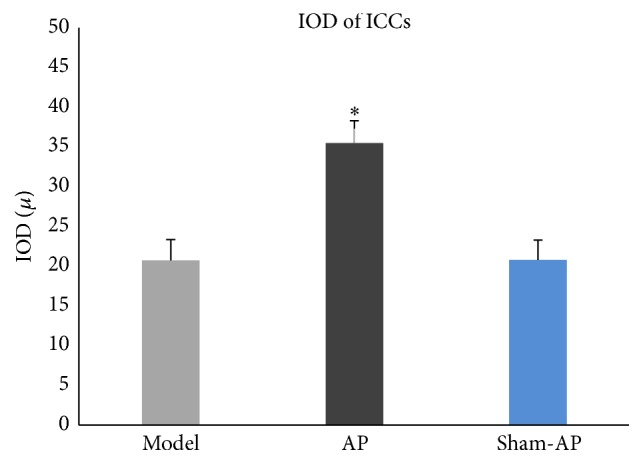
IOD of ICCs. There is significant difference in the IOD of ICCs among three groups (*F* = 187.11, *P* < 0.01). In the AP group, IOD of ICCs is significantly higher than that in the model group (*P* < 0.05), and there is no significant difference between the model group and the sham-AP group. ^*∗*^ *P* < 0.05.

The AP group (Figure 9(b)) shows that the number of ICCs and the synapses of ICCs were significantly increased. ICCs were connected to each other by synapses and formed network structure. The IOD of ICCs was significantly higher than that in the model group (Figure 10).

The sham-AP group (Figure 9(c)) shows that the number of ICCs and the synapses of ICCs were low, and the network integrity was damaged. There was no significant difference in IOD of ICCs between the model group and the sham-AP group (Figure 10).

#### 4.2.4. Serum IgG, IgM, IL-2, and IL-6 Levels (Day 26)

**Figure 11:**
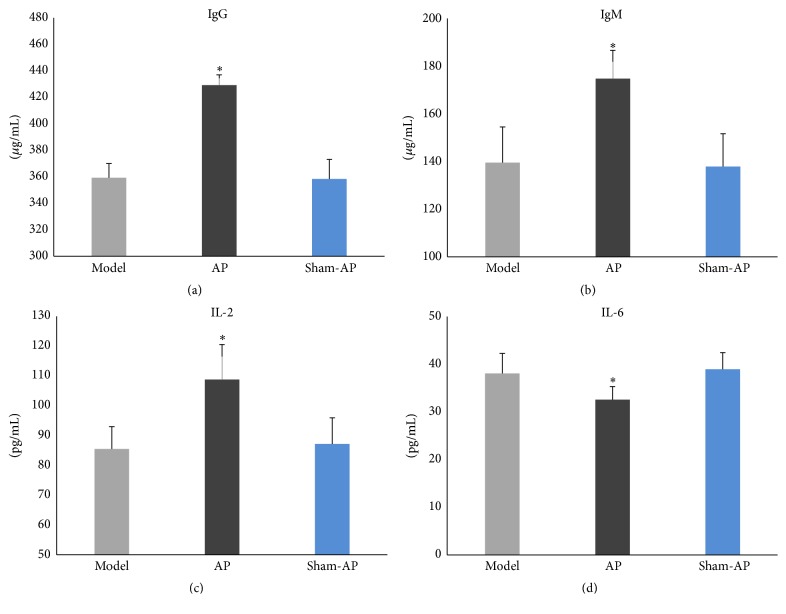
Serum IgG, IgM, IL-2, and IL-6 levels. Whatever the serum IgG, IgM, IL-2, or IL-6 level, there is significant difference among the three groups ((a) *F* = 75.80, *P* < 0.01; (b) *F* = 14.15, *P* < 0.01; (c) *F* = 11.27, *P* < 0.01; (d) *F* = 5.783, *P* < 0.05). In the AP group, the serum IgG, IgM, and IL-2 levels are significantly higher than those in the model group (*P* < 0.05), and the IL-6 level is significantly lower than that in the model group (*P* < 0.05); whatever the serum IgG, IgM, IL-2, or IL-6 level, there is no significant difference between the model group and the sham-AP group. ^*∗*^ *P* < 0.05.

In the AP group, the serum IgG, IgM, and IL-2 levels were significantly higher than those in the model group (Figures 11(a), 11(b), and 11(c)), and the serum IL-6 level was significantly lower than that in the model group (Figure 11(d)). Whatever the serum IgG, IgM, IL-2, or IL-6 level, there was no significant difference between the model group and the sham-AP group.

## 5. Discussion

Nowadays, with the accelerated pace of life, we tend to bear more pressure in our profession and family life. Meanwhile, chronic psychological stress (CPS) related diseases are growing.

CPS can cause various symptoms related to the digestive system. FGID are digestive diseases with high morbidity rates [1, 2], and CPS is a key pathogenic factor [3]. Also, studies have shown that, in CPS patients, there is immune suppression and immune imbalance [5, 6]. At present, its pathogenesis of stress-related diseases has not been studied clearly.

AP, a physical intervention which involves placement of small needles in the skin at different acupoints, has been practiced in China for 2000 years. In the clinic, growing evidences have shown that it could alleviate FGID [7–9]. A randomized, parallel-controlled trial by Shi et al. [8] has shown that, after EA on patients with irritable bowel syndrome, all the symptoms of abdominal pain, diarrhoea, constipation, bloating and flatulence, and vomiting and nausea were significantly improved. A randomized controlled trial by Zhang et al. [9] has shown that for patients undergoing resection of malignant colorectal tumors, after EA on ST36, the time intervals from surgery to the first bowel movement and passage of flatus were shorter than control group patients. Also, studies have shown that AP could improve immunosuppression in patients [10, 11]. A pilot study by Kim et al. [10] has shown that AP may improve the immune system by increasing the counts of a few immune cells and relieve fatigue in cancer patients. A randomized controlled trial by Li et al. [11] has shown that EA appears to reduce immunosuppression of both the humoral and cellular components during surgery.

In this study, CPS rat models were established. Food intake, weight, intestinal sensitivity, ICC of small intestine, and serum immune indexes were measured, and therapeutic effects of EA at ST36 were investigated.

### 5.1. Was the Model Established Successfully?

In clinic, most CPS patients eat less, and some patients show significant weight loss. In this study, food intake and weight were significantly decreased in CPS rats than in the control group, and this is consistent with the clinic. This partially indicates that the models were established successfully.

In clinic, many CPS patients make complaints about the gastrointestinal disorder. In this study, was there abnormality of gastrointestinal function in CPS rats? AWR in response to acute visceral pain was used to assess the intestinal sensitivity following the method of Al-Chaer et al. [23]. The results show that, in CPS rats, scores of AWR were higher significantly. This indicates that there was visceral hypersensitivity in CPS rats. The result partially indicates that the CPS rat models were established successfully.

Studies have shown that CPS is associated with suppression of both cellular and humoral measures [5, 6]. In this study, in order to investigate the immune function, serum IgG, IgM, IL-2, and IL-6 levels were measured. IgG and IgM are the main components of humoral immunity, and serum levels of IgG and IgM are commonly low in immunocompromised patients [25–27]. IL-2 is a very important cytokine for bidirectional immune regulation. Studies have shown that mice deficient in interleukin-2 are very likely to develop autoimmune diseases, for example, inflammatory bowel disease [28, 29]. IL-6 is a proinflammatory cytokine and correlates with immunoreaction closely [30, 31]. In this study, the serum IgG, IgM, and IL-2 levels were lower and the serum IL-6 level was higher in CPS rats than those in control rats. This finding indicates that the immune system may be suppressed and imbalanced in CPS rats. The result partially indicates that the CPS rat models were established successfully.

### 5.2. Therapeutic Effects of EA at ST36

In this study, after EA at ST36, food intake and weight were significantly increased; the scores of AWR were significantly decreased, which indicates the visceral hypersensitivity was improved; the serum IgG, IgM, and IL-2 levels of CPS rats increased markedly, and the serum IL-6 level decreased markedly. It indicates that immune suppression and immune imbalance were improved.

In conclusion, EA at ST36 could improve food intake and weight, could improve the symptom of visceral hypersensitivity, and could regulate immune suppression and immune imbalance in CPS rats. It may be a useful therapeutic approach.

### 5.3. Changes in ICCs in CPS Rats and Therapeutic Effects of EA at ST36

Studies have shown that CPS plays an important role in the development and exacerbation of symptoms in FGID [3]. FGID are relevant to gastrointestinal motility dysfunction [32, 33]. ICCs play an important role in generating and regulating gastrointestinal motility [12]. In this study, in CPS rats, there was visceral hypersensitivity. Also, ICCs in the small intestine lost their normal cellular morphology, and the number was significantly lower than control rats. This may be part of reasons that caused gastrointestinal motility dysfunction of CPS rats.

EA at ST36 showed useful therapeutic effects on visceral hypersensitivity of CPS rats. After treatment, ICCs in intestinal tissues were investigated using confocal laser scanning microscopy, and the results showed that the number of ICCs was increased and cellular morphology resumed normal. This indicated that the mechanisms underlying these effects may be partially related to its repairing effects on ICCs damage.

Studies have shown that brain-gut interactions play an important role in CPS related FGID. The brain receives a constant stream of interoceptive input from the GI, integrates this information with the other interoceptive information from the body and with contextual information from the environment, and sends an integrated response back to various target cells within the GI [34, 35]. In this study, repeated exposure to water avoidance stress will send signals to central nervous system (CNS). Via the neuroendocrine system, the CNS will then send signals back to various target cells within the GI (e.g., ENS). ENS, ICCs, and SMCs connect to form a network structure, which is the basic functional unit of gastrointestinal motility [13–15]. We speculate that the ICCs damage is related to brain-gut interactions. The CPS may increase ICCs apoptosis via the neuroendocrine system, thus causing gastrointestinal motility dysfunction [36].

Studies have shown that neuroendocrine signaling plays a key role in AP treatment. Specifically, AP sends signals to the CNS and the CNS then sends signals to various target cells [37, 38]. We speculate that EA at ST36 may repair ICCs damage by brain-gut interactions. It may promote ICCs proliferation via the neuroendocrine system, thus improving gastrointestinal motility dysfunction [39].

### 5.4. The Relations between Visceral Hypersensitivity and Immunity in CPS Rats

In CPS rats, there were visceral hypersensitivity, immune suppression, and immune imbalance. After EA at ST36, the immune suppression and immune imbalance were improved, and the visceral hypersensitivity was improved either. Studies have shown that visceral hypersensitivity closely correlates with immunity [16–19]. We speculate that the therapeutic mechanisms of EA at ST36 for visceral hypersensitivity may be partially related to its regulation on immune suppression and immune imbalance.

In CPS rats, neuroendocrine signaling may also play an important role in changes in immunity. Repeated stress will send signals to the CNS. Correspondingly, the CNS will send signals to the thymus, spleen, and gastrointestinal tissue via the neuroendocrine system to modulate the immune system. This process is very complex. At present, the hypothalamic-pituitary-adrenal axis, sympathetic nerve-adrenal medulla axis, and some hormones, peptides, and cytokines are thought to be involved [40–42]. In this study, we speculate that CPS may cause immune suppression and immune imbalance which may be part pathogenic factors for visceral hypersensitivity. EA at ST36 may regulate the immunity via neuroendocrine system which is conducive to the improvement of visceral hypersensitivity.

The study indicates that therapeutic mechanisms of EA at ST36 may be multifaceted and target multiple components. Now, the exact mechanisms by which CNS affects ICCs in GI are not fully understood, and the pathophysiologic immunity changes in CPS rats are complex. They remain to be further investigated.

## 6. Conclusion

In CPS rats, EA at ST36 could improve daily food intake and weight as well as the symptom of visceral hypersensitivity. Also, EA at ST36 can regulate immune suppression and immune imbalance. It may be a useful therapeutic approach.

In CPS rats, in small intestine, the morphology of ICCs was abnormal and the number was decreased, which may be part causes of gastrointestinal motility dysfunction. EA at ST36 showed useful therapeutic effects on gastrointestinal motility dysfunction. The mechanisms may be partially related to its repairing effects on ICCs damage.

In CPS rats, there were immune suppression and immune imbalance, which may be part of reasons of visceral hypersensitivity. EA at ST36 showed useful therapeutic effects on visceral hypersensitivity. The mechanisms may be partially related to its regulation on immune suppression and immune imbalance.

## Figures and Tables

**Figure 1:**
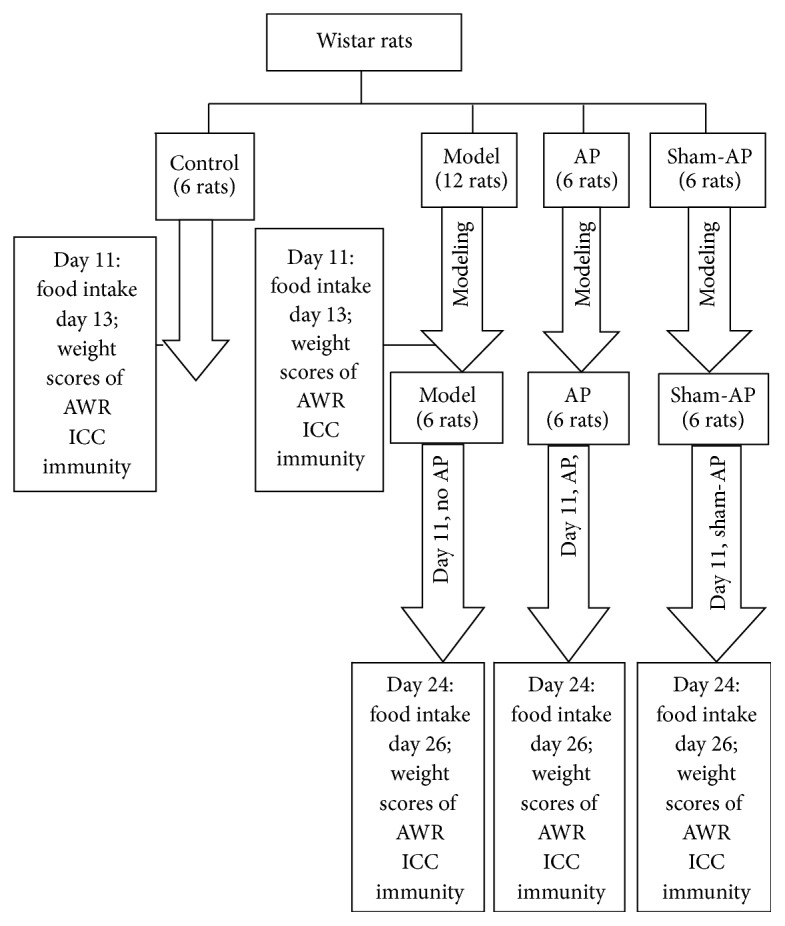
The experimental flow diagram.
